# Youth Group Wellbeing Project for Adolescents Impacted by the March 15 Attacks – Protocol for a Pilot Randomised Waitlist-Controlled Trial

**DOI:** 10.1192/bjo.2024.139

**Published:** 2024-08-01

**Authors:** Katherine Donovan, Shaystah Dean

**Affiliations:** University of Otago, Christchurch, New Zealand

## Abstract

**Aims:**

Psychological distress is common in adolescence; even more so following traumatic events. On 15 March 2019, two mosques in Christchurch were targeted in an act of terrorism. This has had widespread repercussions in the Muslim and wider community in Christchurch and New Zealand. This protocol offers an integrated group treatment based on an indigenous Islamic Psychology framework incorporating components of established transdiagnostic interventions for increasing wellbeing and reducing psychological distress in teenagers. We aim to measure the effect size of the treatment effect on total difficulties, emotional difficulties, trauma symptoms, somatic symptoms and functional impairment in adolescents self-identifying as impacted by the terror attack. We will measure the degree of parental distress and somatic symptoms to explore whether an intervention for adolescents has an impact on parental wellbeing. We will determine the feasibility and acceptability of this approach to inform supports for similar populations and as an example of cultural adaptation of mental health services.

**Methods:**

This is a randomised controlled trial with a waitlist-controlled design to measure the size of treatment effects on clinical outcomes, and the feasibility of this protocol. We aim to recruit 64 participant families. A 6-week group programme will be offered to teenage participants randomised to the study group and offered to the waitlist group following the study. The study will be community-based in one site. We will assess clinical outcomes including emotional difficulties and somatic symptoms in teenagers (aged 12–19) and parents at baseline, end of treatment and at 3-month follow-up, and measure the project's acceptability with participants and parents. Individuals’ experiences of the programme will be examined using qualitative analysis of participant interviews at the end of the programme. Statistical analysis will be a mixed method design including effect size difference calculations, quantitative measures of acceptability and qualitative analysis. Treatment data from participants randomised to the waitlist first will not be included in statistical comparison of treatment effects but will be used for the assessment of feasibility.

**Results:**

This study will inform whether this unique approach is feasible and easily accessible for adolescents impacted by traumatic events. Its design has been driven by community engagement and stakeholder consultation to consider recruitment, relational safety, screening, and risk management. The project has an emphasis on widening access to mental health supports in a minority faith community by maintaining cultural sensitivity and reducing stigma associated with mental illness.

**Conclusion:**

Trial registration: ClinicalTrials.gov, NCT05030909. Registered on 8 September 2021.

